# Estimating metabolic pathway overlap in precision oncology: integrating pharmacogenetics and polypharmacy

**DOI:** 10.3389/fphar.2026.1947359

**Published:** 2026-10-26

**Authors:** Julia C. Stingl, Dominik M. Krzoska, Jule A. Neiss, Jakob Sommer, Thomas Ettrich, Hanna M. Seidling, Thomas Seufferlein

**Affiliations:** 1 Internal Medicine IX - Department of Clinical Pharmacology and Pharmacoepidemiology, Medical Faculty Heidelberg, Heidelberg University Hospital, Heidelberg University, Heidelberg, Germany; 2 Department of Internal Medicine I, Ulm University Hospital, Ulm, Germany

**Keywords:** metabolism, oncology, pathway, pharmacogenetics, phenotype

## Abstract

**Introduction:**

While metabolic pathway overlap is frequent in polypharmacy patients, the modification of genotype-predicted metabolic phenotypes by other substrates is not well established yet, and its relevance for pharmacogenetic implementation in gastrointestinal oncology has not been systematically assessed. We aimed to quantify the overlap between pharmacogenetically relevant treatment pathways and concomitant medications in routine gastrointestinal cancer care.

**Methods:**

Guideline-recommended drugs for gastrointestinal malignancies were evaluated regarding their dependence on pharmacogenetically relevant enzymes. In parallel, real-world co-medication profiles were analyzed to assess the prevalence of inhibitors and inducers of these identified enzymes, as well as the substrate-mediated pathway sharing.

**Results:**

Only a minority of systemic anticancer drugs recommended in treatment guidelines were directly affected by established pharmacogenetic polymorphisms, mainly involving CYP2D6, CYP2C19, CYP2C9, and related pathways. In contrast, co-medication profiles with medications sharing common metabolic pathways were highly prevalent whereas recognized moderate or strong inhibitors were much less frequent (<10%).

**Conclusion:**

Although relatively few anticancer therapies are directly affected by actionable pharmacogenetic variants, the potential for metabolic pathway overlap may increase the clinical relevance of pharmacogenetics in gastrointestinal oncology. By systematically quantifying the coexistence of inhibiting co-medication as well as substrate-mediated pathway sharing, this study provides descriptive explorative estimates of the metabolic pathway-overlap burden. These findings suggest that pharmacogenetic implementation strategies should consider both genotype and medication-related pathway overlap to better inform estimates of drug exposure and support precision oncology.

## Introduction

### Pharmacogenetics as a tool for precision dosing in oncology

Comprehensive genomic profiling has become routine in oncology to identify actionable somatic alterations and guide personalized treatment decisions (Campani et al., 2026). In addition to tumor-specific mutations, these analyses frequently generate information on germline genetic variants, including pharmacogenetic polymorphisms. Although such data are often available, they are rarely incorporated into routine clinical decision-making. This may partly reflect the fact that many modern targeted anticancer therapies are only modestly affected by common pharmacogenetic variants (Nogueiras-Álvarez and Pérez Francisco, 2024). Consequently, the direct contribution of pharmacogenetics to drug selection in oncology appears limited compared with its role in other therapeutic areas.

Recent bioinformatics-driven advances in pharmacogenomics have enabled increasingly sophisticated interpretation of genomic data through functional variant annotation, computational phenotype prediction, and integration of diverse pharmacogenomic resources (Zaid et al., 2026). Modern pharmacogenomic pipelines now support the translation of genomic information into clinically actionable recommendations and represent an important component of precision medicine (Lauschke et al., 2024). Nevertheless, most current approaches focus primarily on genotype-derived phenotype prediction and only incompletely account for dynamic modifiers of enzyme activity occurring during treatment. In particular, the integration of pharmacogenetic information with drug-drug interactions and phenoconversion remains insufficiently addressed, despite its potential importance for estimating actual metabolic capacity in oncology patients receiving complex medication regimens (Stingl and Viviani, 2025).

Nevertheless, pharmacogenetics remains highly relevant for optimizing drug exposure and tolerability, particularly in treatment settings where achieving adequate dose intensity is critical for therapeutic success. Consequently, individual tolerance of treatment-related adverse effects may be crucial for maintaining treatment intensity and achieving favorable outcomes. However, safety is not solely determined by the intrinsic toxicity profile of the anticancer drug but also by interindividual differences in drug metabolism, co-medication, and drug–drug interactions. Variability in exposure may therefore contribute to treatment-limiting toxicity, dose reductions, treatment delays, or premature discontinuation, all of which can compromise treatment efficacy (Mohamed et al., 2023).

### Pharmacogenetics and phenoconversion

The metabolic phenotype of an individual is determined not only by inherited genetic variation but also by environmental factors, particularly concomitant medication. As discussed previously, pharmacogenetics therefore has a dual role: genetic variants define baseline metabolic activity, whereas drug–drug interactions may substantially modify enzyme function and alter the clinically expressed phenotype (Derobertmasure et al., 2025).

This phenomenon, termed phenoconversion, describes the situation in which the actual metabolic capacity deviates from the genetically predicted phenotype due to enzyme inhibition or induction. For major pharmacogenetically actionable pathways, such as CYP2D6, CYP2C19, and CYP2C9, drug-induced changes in enzyme activity may be quantitatively comparable to the differences observed between pharmacogenetic groups. In the present study, a pathway was considered pharmacogenetically actionable if prescribing recommendations based on genotype-defined metabolizer status are provided by the summary of product characteristics or by implementation consortia including the Clinical Pharmacogenetics Implementation Consortium (CPIC) or the Dutch Pharmacogenetics Working Group (DPWG). Consequently, a genetically normal metabolizer may functionally exhibit an intermediate or poor metabolizer phenotype under the influence of interacting medications.

As illustrated in Figure 1, increasing inhibition by drug-interactions progressively reduces enzyme activity toward poor-metabolizer levels. The magnitude of this shift depends on the genetically determined baseline activity, such that identical inhibitory or inductive effects may result in different clinically relevant phenotype transitions. Thus, incorporation of phenoconversion may be viewed as an important extension of current pharmacogenomic interpretation pipelines, enabling a closer approximation of real-world drug metabolism than genotype-based prediction alone. Phenoconversion is used as term for the change in drug metabolizer phenotype mediated by recognized inhibitors and inducers while substrate-only metabolic pathway overlap does not constitute evidence of phenoconversion. Although phenoconversion as a modifier of genotype-predicted drug metabolism is a well-recognized concept, the field still lacks quantitative approaches capable of estimating the extent of enzyme phenotype alterations by substrates that share the same metabolic pathway, particularly in oncology. To overcome this limitation, we recently proposed a quantitative model that translates substrate-associated pathway-overlap into estimates of actual CYP enzyme activity and corresponding metabolic phenotypes (Stingl et al., 2026). This approach expands the current phenoconversion framework beyond simple identification of recognized interacting drugs and provides a quantitative basis for assessing metabolic variability in oncology patients exposed to complex medication regimens. Although interactions are influenced by multiple factors, including substrate affinity, inhibitor potency, dose, exposure, timing, transporter activity, and enzyme reserve capacity, our quantitative model showed that integration of the net effect of these determinants into a single phenotype estimate was feasible, and none of these factors emerged as significant independent predictors beyond the quantitative phenotype estimate itself (Stingl et al., 2026).

**FIGURE 1:**
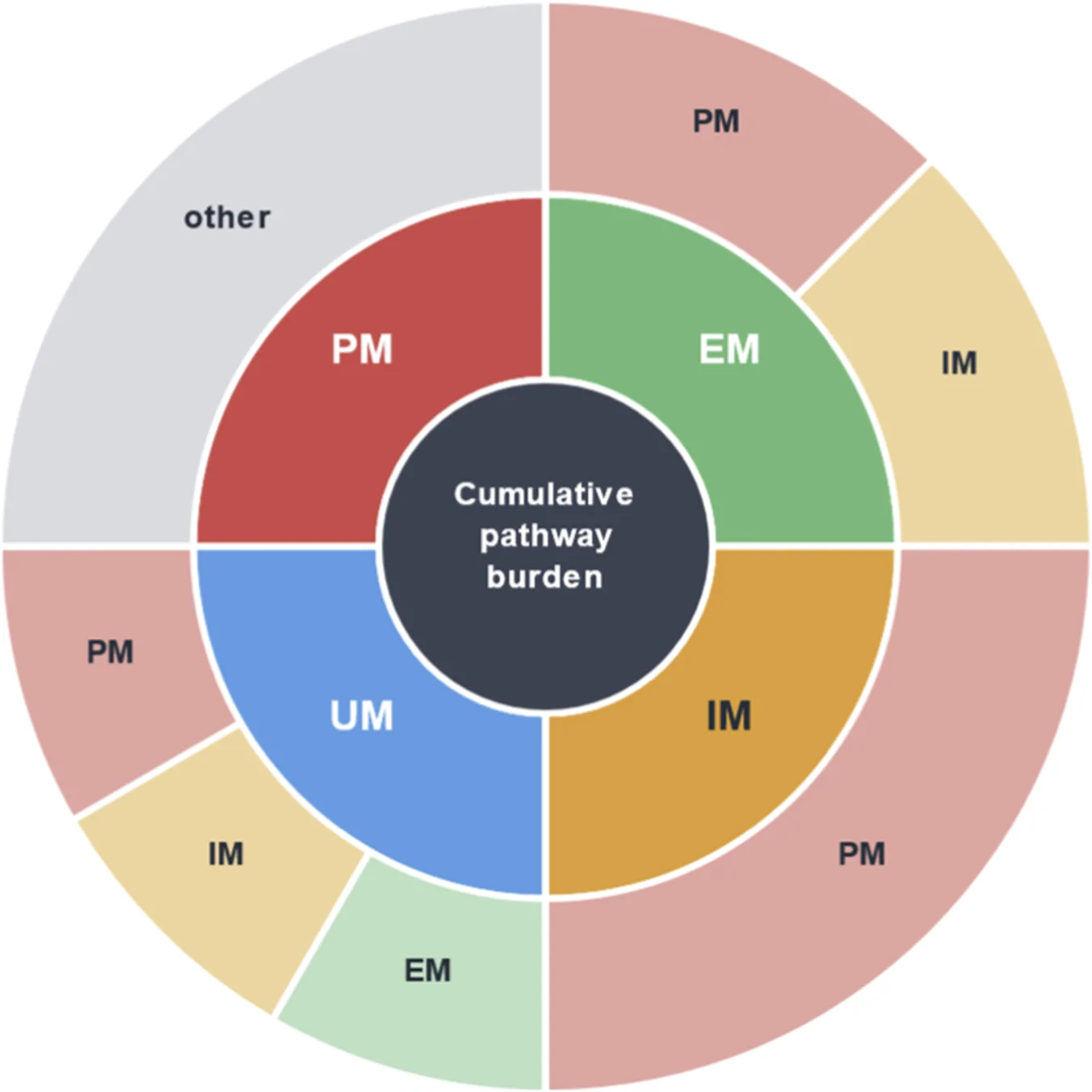
Biological interpretation of phenoconversion as the combined effect of inherited enzyme activity and medication-induced modulation of drug metabolism. The inner circle depicts genotype-predicted metabolizer phenotypes (PM, IM, EM, UM), whereas the outer circle represents the clinically expressed phenotype after considering inhibitory or inducing co-medications. Drug-induced changes in enzyme activity may shift patients across metabolizer categories, creating a mismatch between genotype-predicted and actual metabolic capacity. This concept highlights that drug exposure and treatment response may be determined not only by genetic variation but also by the cumulative burden of concomitant medications acting on the same metabolic pathway.

### Polypharmacy as a major determinant of drug exposure in cancer patients

Polypharmacy is highly prevalent among cancer patients, particularly among older individuals and those receiving supportive care medications (Zweers-Zeilmaker et al., 1997; Sokol et al., 2007; Jerjes, 2026; Tawengi et al., 2026). In addition to anticancer treatment, patients frequently receive analgesics, antiemetics, proton pump inhibitors, anticoagulants, antihypertensives, antidepressants, and other chronic medications. As a result, drug–drug interactions represent a major determinant of drug exposure and treatment tolerability in routine oncology practice.

Several studies have demonstrated clinically relevant associations between concomitant medication and treatment-related toxicity (Mohamed et al., 2023). For example, polypharmacy has been associated with increased irinotecan-related adverse drug reactions, while concomitant medications have also been shown to modify toxicity risks during immune checkpoint inhibitor therapy (Sasaki et al., 2013; Miao and Herrmann, 2023). Such observations suggest that the consequences of phenoconversion may be particularly relevant for anticancer therapies because many oncological drugs have a narrow therapeutic window. Small changes in drug clearance can therefore lead to clinically meaningful differences in systemic exposure, influencing both toxicity and treatment efficacy.

We have shown that in case of polypharmacy, metabolic pathway overlap by substrates may shift the phenotype of CYP2D6 (Saromba et al., 2025) and CYP2C19 (Stingl et al., 2026). Here, we will look at both mechanism modifying genotype-predicted drug metabolism, and explore its potential impact on precision oncology. In patients with gastrointestinal cancers, who are frequently exposed to complex treatment regimens and extensive polypharmacy, the extent to which concomitant medications may alter pharmacogenetically relevant metabolic pathways by either mechanism remains largely unknown.

We hypothesize that pharmacogenetically relevant pathways frequently overlap with concomitant medications capable of modifying pathway activity in gastrointestinal oncology. Therefore, we aim to quantify both, pathway overlap by substrates and phenoconversion by inhibitors or inducers in routine cancer care by evaluating:

- the proportion of guideline-recommended systemic therapies that rely on pharmacogenetically actionable metabolic pathways (“victim drug burden”), and
- the prevalence of concomitant medications with the capacity to either inhibit or induce these pathways or modify predicted metabolizer status by substrate pathway overlap (“pathway overlap burden”).

## Methods

### Assessment of the metabolic pathways in oncological drugs - as use case here: drug therapy in gastrointestinal cancer as “victim drugs” for drug-interactions

A structured assessment of the clinical practice guidelines for gastrointestinal malignancies was conducted to identify drugs recommended in treatment. Guideline-based treatment regimens were extracted from the current Onkopedia guidelines of the German Society for Hematology and Medical Oncology (DGHO), which provide evidence-based recommendations for the diagnosis and treatment of hematologic and oncologic diseases and are regularly updated by multidisciplinary expert panels (Lordick et al., 2025; Reichardt et al., 2026; Sinn et al., 2024; Stintzing et al., 2025; Hofheinz et al., 2026a; Hofheinz et al., 2026b; Engelhardt et al., 2023). All identified compounds were entered into a structured database for further analysis. For each drug, detailed information on metabolic pathways was retrieved from the corresponding German summary of Product Characteristics (SmPC). This included the identification of enzymes involved in biotransformation, as well as relevant drug transporters contributing to absorption, distribution, and elimination. Furthermore, the relative contribution of each metabolic pathway was categorized (e.g., major versus minor) according to the classifications provided in the product information. Pharmacogenetic actionability was defined *a priori* at the enzyme level. Enzymes were classified as pharmacogenetically actionable when clinically implemented dosing or prescribing recommendations based on genotype-predicted metabolizer status were available in CPIC or DPWG guidelines. Accordingly, CYP2D6, CYP2C19, CYP2C9, DPYD, UGT1A1 were considered actionable pathways, whereas pathways without guideline-supported clinical implementation recommendations were not categorized as actionable, irrespective of their quantitative contribution to drug metabolism.

In addition, treatment regimens recommended in the guidelines that comprise fixed combinations of agents, such as standard chemotherapy protocols, were separated and systematically analyzed drug by drug. For each drug, the overall metabolic pathway burden was derived.

### Data analysis of polypharmacy profiles

To characterize inhibition, induction or substrate-mediated pathway overlap in the context of polypharmacy, a second analytical step focused on the derivation of “typical” co-medication profiles. Polypharmacy profiles usually depict long-term medication for non-communicable diseases or symptoms. Polypharmacy data of patients are available from the ADRED study, a large prospective multicenter cohort including more than 8,000 patients presenting in emergency visits with adverse drug reactions (Schurig et al., 2018; Just et al., 2020a).

Given that many recorded events were attributable to the cumulative effects of polypharmacy rather than to a single compound, the ADRED dataset provides a representative overview of commonly co-administered medications in an elderly, multimorbid population (Just et al., 2020b; Just et al., 2020c). In the ADRED cohort, one study center was a gastrointestinal/oncology specialized emergency unit and due to a higher prevalence of oncological patients was analyzed separately and compared with the overall cohort to identify frequent co-occurring medication patterns. Rather than evaluating individual drugs in isolation, each patient case was transformed into a cumulative “pathway burden” profile. To this end, all recorded medication per case were systematically annotated with their interaction-relevant properties, including substrate, inhibitor, and inducer characteristics for drug-metabolizing enzymes and transporters. The substrate, or inhibitor or inductor properties for each drug were taken from two sources: (1) from the FDA list of Drugs that Interact with CYP Enzymes and Transporter Systems and (2) An extraction was performed primarily from Section 5.2, “Pharmacokinetic properties”, of the corresponding German Summary of Product Characteristics (“Fachinformation”) (U.S. FoodD. Administration, 2026). This was achieved using a two-step LLM-assisted pipeline. In Step 1, the model was prompted to extract information on unchanged elimination and CYP-mediated metabolism from raw German SmPC HTML text and to return the results as a structured Markdown summary. In Step 2, a second prompt was used to convert this Markdown summary into a predefined JSON schema validated with Pydantic. To enable reproducibility, the complete Python script is available in the associated BWGit repository: URL: https://bwgit.de/hd-vy353/Phenoconversion_Precision_Oncology. Full prompts are provided in the Supplementary Material (Supplementary Code, Supplementary Prompt).

The validation sample was intended as a pragmatic quality-control assessment of the extraction workflow rather than a formal performance evaluation of the underlying language model. To evaluate the pipeline, 50 drugs were selected at random from the overall dataset to capture a heterogeneous set of SmPC structures and metabolic pathway descriptions resulting in an error rate of 6.0% (95% CI 2.1%–16.2%). All identified discrepancies were attributable to incomplete extraction or misclassification of individual pathway annotations rather than systematic failure of the extraction workflow. No recurring error pattern suggesting bias towards specific CYP enzymes was observed within the validation sample.

Two reviewers independently extracted information on unchanged elimination and CYP-mediated metabolism from the relevant SmPCs. Following the extraction criteria specified in the prompt, the reviewers primarily assessed Section 5.2 in the SmPC, recording information on the proportion excreted unchanged and the involvement of CYP enzymes. Where no explicit numerical value was reported, qualitative expressions were translated into predefined semi-quantitative estimates as unvalidated ordinal coding convention. “Complete” or “exclusive” was assigned a value of 1.0, “mainly” or “predominantly” a value of 0.8, “half” a value of 0.5, “partially” or “moderately” a value of 0.3, and “minor” or “to a small extent” a value of 0.1. Pathways with a semiquantitative estimate greater than 0.5 were subsequently classified as major pathways. These semiquantitative weights were introduced because SmPCs frequently describe pathway contributions qualitatively rather than numerically. The assigned values do not represent experimentally measured fractions metabolized but provide a standardized approximation enabling comparative pathway-level analyses across drugs. The weighting scheme preserves the ordinal information contained in regulatory product information and was applied uniformly to all substances analyzed. Where available, numerical values and supporting quotations were extracted. The reviewers were blinded to the outputs of the pipeline. Any disagreements were resolved by consensus to create a manual reference standard. Inter-reviewer agreement was assessed using the percentage of agreed cases. The pipeline outputs were then compared with the reference standard, and the error rates were calculated with 95% confidence intervals using the Wilson score interval.

### Analysis of victim drugs and metabolic pathway overlap

The unit of analysis differed between the victim and co-medication assessments. For victim burden, the primary unit of analysis was the individual anticancer drug (n = 35 drugs extracted from guideline-recommended regimens). For pathway overlap burden, the primary unit of analysis was the patient co-medication profile. Frequencies of pathway involvement among medications were summarized at the level of unique active substances (n = 839 drugs in the n = 7967 ADRED cases). To estimate the potential pathway sharing in oncological drug therapy, an integrated pathway-based analysis of medication was performed. Victim burden was defined as the relative dependence of guideline-recommended anticancer therapies on a specific metabolic or transporter pathway. Pathway overlap burden was defined as the frequency with which concomitant medications within a patient cohort were capable of interacting with the same pathway as substrates.

First, the victim burden of guideline-recommended oncological therapies was characterized. For each individual drug and treatment regimen, all relevant drug-metabolizing enzymes and transporters involved in absorption, distribution, metabolism, and elimination were identified. Pathway burden was quantified by summing the fractional contribution (= the contribution of each metabolizing enzyme to a drug metabolic pathway) across all analyzed drugs, thereby determining the relative importance of individual metabolic and transport pathways within guideline-based oncological treatment regimens. The relative contribution to each metabolic pathway was categorized (e.g., major versus minor) according to the classifications provided in the product information.

Second, the metabolic pathway overlap was evaluated by assessing the prevalence of pathway-modifying co-medications in real-world polypharmacy profiles. Because cancer patients frequently present with polypharmacy at the time of diagnosis and during treatment, medication profiles from the ADRED cohort were used as a representative for patients with polypharmacy and clinically relevant drug-interaction patterns associated with adverse drug reactions. The ADRED cohort was not designed as an oncology-specific cohort and was therefore used primarily as a representative source of real-world polypharmacy profiles and pathway overlap patterns. Consequently, individual anticancer treatment regimens were not linked to specific patient medication profiles. The objective of the present study was not to quantify treatment-specific drug-drug-gene interactions but to estimate the frequency with which pharmacogenetically relevant metabolic pathways are simultaneously represented among guideline-recommended gastrointestinal cancer therapies and typical polypharmacy profiles. To explore potential differences, the cohort was stratified into oncological-enriched and non-oncological patient subgroups.

For each patient profile, all medications were classified according to their role as substrates, inhibitors, or inducers of the respective drug-metabolizing enzymes and transporters.

Pathway involvement was determined from the respective German Summary of Product Characteristics (SmPC). Where available, the quantitatively reported contribution of the pathway to the drug’s metabolism was used, and if no quantitative information was provided, pathway involvement was conservatively classified as minor. The medication profiles involving multiple drugs were quantified by aggregating the involvement of pathways, without modelling specific drug interactions separately. Accordingly, the analysis was performed at the pathway level rather than at the individual drug-interaction level. The objective was to estimate the frequency with which clinically relevant metabolic pathways are shared within polypharmacy settings. The frequency of pathway involvement was then calculated across all medication profiles.

## Results

### Analysis of oncological drugs as victims of pathway sharing in polypharmacy

Current guideline-recommended therapies for gastrointestinal malignancies were extracted from the respective Onkopedia guidelines. All identified small-molecule drugs and fixed-dose combinations were entered into a structured database for further analysis, whereas monoclonal antibodies and other protein-based therapeutics were excluded because their elimination is generally independent of cytochrome P450-mediated metabolism. For each drug, information on metabolic pathways and the relative contribution of individual enzymes was extracted from the respective SmPC.

A total of 35 drugs were identified from the guidelines and included in the analysis (Table 1). For each compound, drug-metabolizing enzymes and transport pathways involved in clearance were classified according to their reported contribution as explained above.

**TABLE 1:** Metabolic pathways of small-molecule drugs recommended for the treatment of gastrointestinal malignancies.

Drug	Metabolized	Metabolism by
5-Fluorouracil	Major	DPYD
Apatinib (Rivoceranib)	Major	CYP3A4
​	Minor	CYP2D6
​	Minor	CYP2C9
​	Minor	CYP3A5
Avapritinib	Major	CYP3A4
​	Major	CYP3A5
​	Minor	CYP2C9
​	Minor	UGT1A3
Cabozantinib	Major	CYP3A4
​	Minor	CYP2C9
Capecitabin	Major	DPYD
Carboplatin	-	No CYP enzyme involvement
Cisplatin	-	No CYP enzyme involvement
Docetaxel	Major	CYP3A4
Encorafenib	Major	CYP3A4
​	Minor	CYP2C19
​	Minor	CYP2D6
Entrectinib	Major	CYP3A4
​	Minor	UGT1A4
Erlotinib	Major	CYP3A4
​	Minor	CYP1A2
​	Minor	CYP1B1
​	Minor	CYP1A1
Etoposid	Minor	CYP3A4
Everolimus	Major	CYP3A4
Folinic acid	-	No CYP enzyme involvement
Fruquintinib	Minor	CYP3A4
​	Minor	CYP2C8
​	Minor	CYP2C9
​	Minor	CYP2C19
Futibatinib	Minor	CYP3A4
Gemcitabine	-	No CYP enzyme involvement
Imatinib	Major	CYP3A4
Irinotecan	Major	CYP3A4
​	Minor	CYP3A5
​	Major	UGT1A1
Ivosidenib	Major	CYP3A4
Larotrectinib	Major	CYP3A4
​	Major	CYP3A5
Lenvatinib	Major	CYP3A4
Mitomycin	-	No CYP enzyme involvement
Oxaliplatin	-	No CYP enzyme involvement
Paclitaxel	Major	CYP2C8
​	Major	CYP3A4
Pemigatinib	Major	CYP3A4
Regorafenib	Major	CYP3A4
​	Minor	UGT1A9
Ripretinib	Major	CYP3A4
​	Major	CYP3A5
​	Minor	CYP2C8
​	Minor	CYP2D6
Selpercatinib	Major	CYP3A4
Sorafenib	Minor	CYP3A4
​	Minor	UGT1A9
Streptozocin	-	No CYP enzyme involvement
Sunitinib	Major	CYP3A4
Tegafur/Gimeracil/Oteracil	Major	DPYD
​	Major	CYP2A6
Trifluridin/Tipiracil	-	No CYP enzyme involvement
Tucatinib	Major	CYP2C8
​	Minor	CYP3A4

The contribution of individual metabolic pathways was aggregated across all included drugs. Overall, 16 distinct drug-metabolizing enzymes and transport-related pathways were identified. CYP-mediated metabolism accounted for the majority of pathway involvement, with CYP3A4/5 representing by far the most frequently utilized metabolic pathway. The CYP3A4 enzyme is encoded by the *CYP3A4* gene, but currently known *CYP3A4* genetic variants do not produce the clearly separated metabolizer phenotypes (Matthaei et al., 2020). There are few variants that decrease the function of the enzyme but they are rare (CYP3A4*22, minor allele frequency of 3%–5%). Consistently, no current CPIC or DPWG guideline recommends routine *CYP3A4* genotyping (Mulder et al., 2021).

Other relevant pathways included CYP1A2, CYP2C8, CYP2C9, CYP2C19, and CYP2D6 (Shimada et al., 1994; Achour et al., 2014), whereas phase II enzymes and transporter pathways contributed only to a limited extent.

Importantly, only a subset of these pathways is characterized by clinically actionable pharmacogenetics. Consequently, the direct impact of pharmacogenetic polymorphisms on guideline-recommended therapies for gastrointestinal cancers appears limited, affecting only drugs whose disposition substantially depends on enzymes encoded by pharmacogenetically actionable genes. However, many of these drugs depend on CYP pathways that are highly susceptible to inhibition or induction by concomitant medication. Therefore, although classical drug–gene interactions are comparatively infrequent in our analysis, the potential for drug–drug–gene interactions is substantial.

The resulting pathway distribution is shown in Figure 2 and reflects the relative “victim burden” of metabolic pathways in guideline-based gastrointestinal cancer therapy. The predominance of CYP3A-mediated metabolism is consistent with the overall contribution of CYP3A enzymes to drug metabolism in clinical pharmacology and indicates that phenoconversion through CYP3A inhibition or induction may affect a large proportion of oncological therapies despite the relatively limited number of directly pharmacogenetically actionable drugs.

**FIGURE 2:**
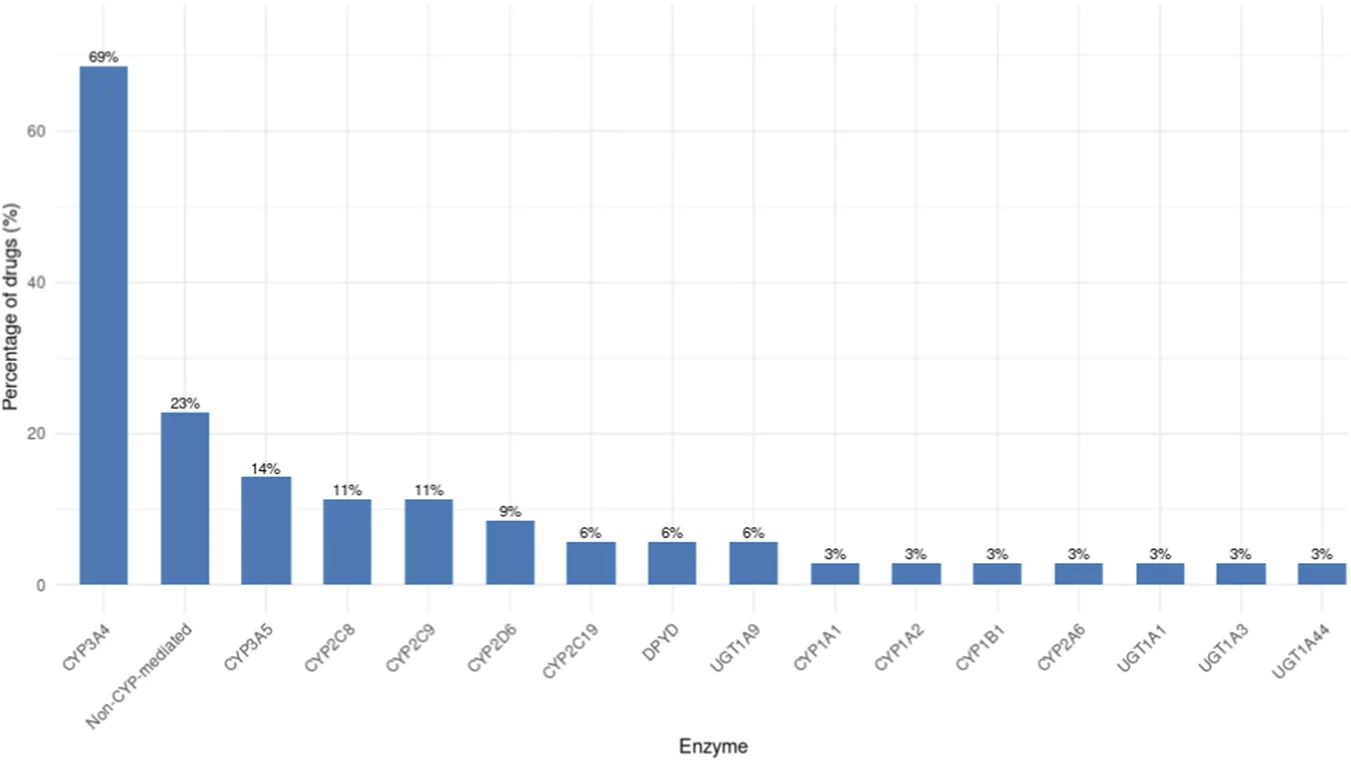
Relative involvement of metabolic pathways among guideline-recommended small-molecule therapies for gastrointestinal cancers (n = 35). The figure represents the “victim-drug burden”, indicating the proportion of anticancer therapies potentially affected by alterations in specific metabolic pathways. While only a subset of pathways is associated with actionable pharmacogenetic variation, the predominance of CYP3A4 suggests that many therapies may be vulnerable to medication-induced changes in enzyme activity. The observed distribution therefore provides a biological basis for estimating the potential impact of phenoconversion in gastrointestinal oncology, as concomitant inhibitors or inducers may modify metabolic activity independently of genotype.

### Analysis of the metabolic pathway overlap in polypharmacy

To estimate the potential pathway overlap in real-world polypharmacy, medication profiles from the ADRED cohort were analyzed. The dataset comprised 7,967 patient cases presenting with adverse drug reactions and included a median of seven concomitantly administered drugs per patient. Overall, 839 distinct active substances were identified across all medication profiles.

All small-molecule drugs recorded in the cohort were systematically evaluated with regard to their involvement in drug-metabolizing pathways. For each compound, substrate, inhibitor, and inducer characteristics were assigned to the corresponding metabolic enzymes. The frequency of pathway involvement was then aggregated across all identified medications.

Regarding the LLM-assisted extraction pipeline, the reviewers agreed on 49 of 50 assessments, corresponding to an inter-reviewer agreement of 98%. Compared with the consensus-based manual reference standard, the pipeline produced 3 errors among 50 evaluated outputs, resulting in an error rate of 6.0% (95% CI 2.1%–16.2%).

Among the 839 compounds, CYP3A was the most frequently involved metabolic pathway, followed by CYP2D6, CYP2C19, and CYP2C9 (Supplementary Figure S1). Thus, the pathways most commonly represented among the substance largely overlapped with the pathways identified for oncological victim drugs, with no statistically significant differences observed for six of the seven CYP enzymes. CYP3A was the exception, being significantly more prevalent among oncological victim drugs than among the co-medication substrates (68.6% vs. 29.4%; OR 5.23; p < 0.001; Supplementary Table S4, Supplementary Material).

When looking at individual cases, the distribution of pathway involvement among concomitant medications is shown in Figure 3b and Table 2. As can be seen, the relative frequency to have one or two or more substrates metabolized by CYP3A in the co-medication is 84% (one) or 64% (>=two). The relative frequency to have at least one substrate for a polymorphic enzyme (CYP2D6, CYP2C19 or CYP2C9) is more than 50% (57%, 54%, 40%). To explore the robustness of the findings, analyses were additionally examined in the oncology-enriched subgroup of patients from the oncology-focused study center in Ulm University, (Figure 3b, orange). This subgroup showed a comparable or higher burden of pathway overlap than the overall cohort, with 93% of CYP3A, and 69%, 72% and 44% for CYP2D6, CYP2C19 and CYP2C9, supporting the relevance of the findings for oncology patients. These descriptive differences were statistically detectable for CYP3A, CYP2D6 and CYP2C19 after adjustment for multiple testing (p < 0.001 for CYP3A, CYP2D6 and CYP2C19), but not for CYP2C9 (Holm-adjusted p = 0.083). Complete estimates, 95% confidence intervals and test results are provided in Supplementary Table S3.

**FIGURE 3:**
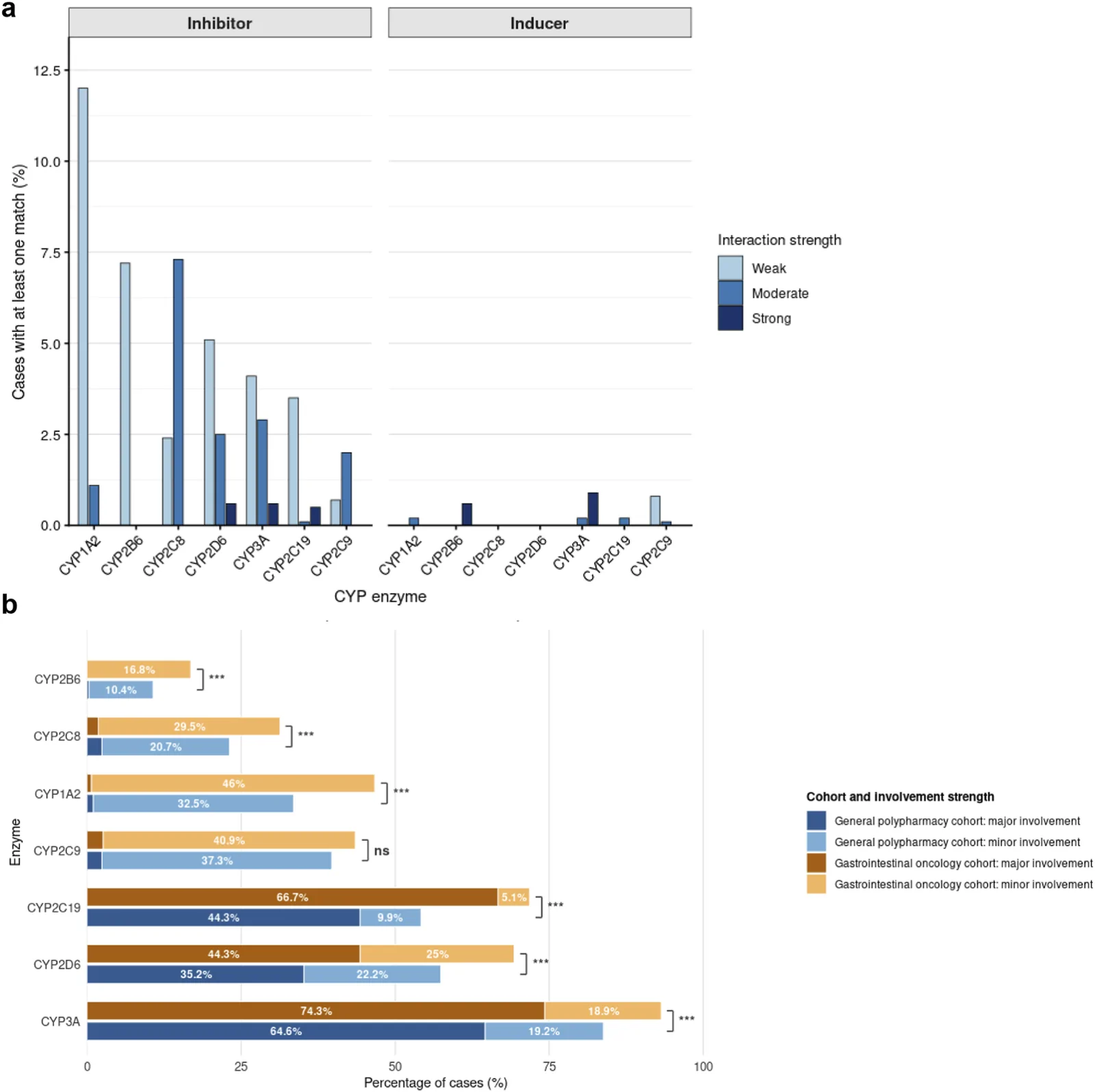
**(a)** Perpetrator burden by recognized inhibitors and **(b)** patient-level pathway overlap burden in the ADRED cohort. **(a)** Distribution of CYP enzyme inhibitors and inducers among all reported medications (n = 839 distinct compounds), illustrating the availability of co-medications capable of modifying enzyme activity and thereby contributing to phenoconversion potential. **(b)** Proportion of patients exposed to drugs involving the indicated metabolic pathways. Major and minor involvement reflect the relative contribution of each pathway to drug metabolism. Together, both panels demonstrate that pathways frequently used by medications in patients with polypharmacy are also commonly subject to enzymatic inhibition or induction, providing a biological basis for a high phenoconversion potential in routine clinical care. Brackets indicate comparisons of total CYP involvement between cohorts. *** describe a multiplicity-adjusted p-value <0.001; ns, not significant.

**TABLE 2:** Characteristics of the ADRED study population and prevalence of medications involving cytochrome P450 enzymes, stratified by study cohort.

Total ADRED study: n = 7,967	General polypharmacy cohort (all other study sites): n = 7,400	Gastrointestinal/oncology cohort (study site Ulm): n = 567 (47% oncological patients)
Median number of drugs taken (Q1 - Q3)	N = 7 (Lauschke et al., 2024; Stingl and Viviani, 2025; Mohamed et al., 2023; Derobertmasure et al., 2025; Stingl et al., 2026; Zweers-Zeilmaker et al., 1997; Sokol et al., 2007; Jerjes, 2026)	N = 10 (Derobertmasure et al., 2025; Stingl et al., 2026; Zweers-Zeilmaker et al., 1997; Sokol et al., 2007; Jerjes, 2026; Tawengi et al., 2026; Sasaki et al., 2013)
Patient sex (Percentage female)	49.6%	44.3%
Mean age (years)	68.4	68.1
Percent patient cases with at least one involved drug for the respective CYP enzyme:		
CYP1A2 (major/minor)	1.0%/32.5%	0.7%/46.0%
CYP1A2 (2 drugs or more)	7.2%	12.5%
CYP3A (major/minor)	64.6%/19.2%	74.3%/18.9%
CYP3A (2 drugs or more)	64.2%	79.7%
CYP2B6 (major/minor)	0.3%/10.4%	0.0%/16.8%
CYP2B6 (2 drugs or more)	0.2%	0.0%
CYP2C9 (major/minor)	2.4%/37.3%	2.6%/40.9%
CYP2C9 (2 drugs or more)	9.8%	9.3%
CYP2C8 (major/minor)	2.4%/20.7%	1.8%/29.5%
CYP2C8 (2 drugs or more)	3.5%	6.2%
CYP2C19 (major/minor)	44.3%/9.9%	66.7%/5.1%
CYP2C19 (2 drugs or more)	19.0%	28.2%
CYP2D6 (major/minor)	35.2%/22.2%	44.3%/25.0%
CYP2D6 (2 drugs or more)	23.2%	30.7%

FDA-labeled CYP enzyme-inducing co-medication was uncommon in both cohorts, with observed proportions of approximately 1% or less irrespective of the CYP enzyme considered (Figure 3a; Supplementary Table S1). In contrast, mechanism-based CYP enzyme inhibitors were more frequently observed. In the general polypharmacy cohort, the highest proportions were observed for CYP1A2 inhibitors (12.9%), CYP2C8 inhibitors (9.0%), CYP2D6 inhibitors (8.1%), and CYP3A inhibitors (6.8%). In the gastrointestinal oncology cohort, inhibitor proportions were descriptively higher for several enzymes, particularly CYP2C8 (17.6%), CYP3A (15.9%), CYP1A2 (15.5%), and CYP2B6 (12.5%).

The presence of a substrate for the respective enzyme was statistically detectable for all investigated enzymes and was observed irrespective of concomitant administration of inhibiting or inducing co-medication (p < 0.001, Supplementary Table S5, Supplementary Material). Thus, when it comes to polypharmacy, the prevalence of drugs sharing or modifying pharmacogenetically relevant pathways is very high.

So, in general, co-medication involving shared substrates is much more prevalent than co-medication involving established inhibitors or inducers. Interestingly, as can be seen in Figure 3b), most of the substrate of CYP3A4, CYP2D6 and CYP2C19 were major substrates metabolized predominantly by this enzyme. For CYP2C9, CYP2C8 and the other CYPs, involvement in metabolism is usually in-between other enzymes, leading only to a minor pathway occupation.

## Discussion

While polypharmacy is frequent in cancer patients, relatively few anticancer therapies are directly affected by actionable pharmacogenetic variants. By combining analyses of anticancer drug metabolism with pathway analysis of polypharmacy profiles from a real-world cohort, our study provides an estimate of the overlap between pharmacogenetically relevant metabolic pathways. To estimate clinically relevant polypharmacy patterns, we analyzed medication profiles from patients who experienced adverse drug reactions leading to emergency department presentations, a cohort that is likely enriched for clinically significant drug interactions and adverse outcomes (Just et al., 2020a).

The denominator for victim burden consisted of 35 guideline-recommended anticancer drugs, whereas the pathway overlap was derived from 7,967 patient medication profiles (ADRED case size) and 839 distinct active substances. Consequently, the reported estimates should be interpreted as patient-level estimates of pathway overlap frequency. Because the ADRED cohort exclusively comprises patients with ADRs, selection bias cannot be excluded. However, it was justified because the primary objective of the present analysis was not to estimate the prevalence of co-medication with pathway overlap in the general oncology population but rather to investigate its occurrence and potential clinical relevance in patients experiencing ADRs, a setting in which sharing of drug metabolism pathways is particularly likely to contribute to adverse outcomes. From this perspective, the enrichment for ADR-related cases represents a strength, as it increases the probability of identifying clinically meaningful pathway overlap in drug metabolism. Nevertheless, the resulting frequencies should be interpreted as estimates within an ADR population and not be directly extrapolated to unselected oncology cohorts.

Based on these data, we demonstrate that more than 80% of polypharmacy profiles contain medications sharing metabolic pathways relevant to oncological therapies. Such pathway overlap may contribute to substrate competition, thereby creating the conditions of altering the patient’s actual drug metabolism phenotype. The degree of altering the phenotype is unclear, but recent analyses revealed a shift in enzyme activity of about 30% by a major substrate in the case of CYP2C19 (Stingl et al., 2026). The mechanistic chain from germline genotype through baseline enzyme activity and pathway sharing by co-medication, and its possible downstream implications for drug exposure and clinical outcome are summarized in Figure 4, using CYP2C19 and escitalopram as a well characterized illustrative example.

**FIGURE 4:**
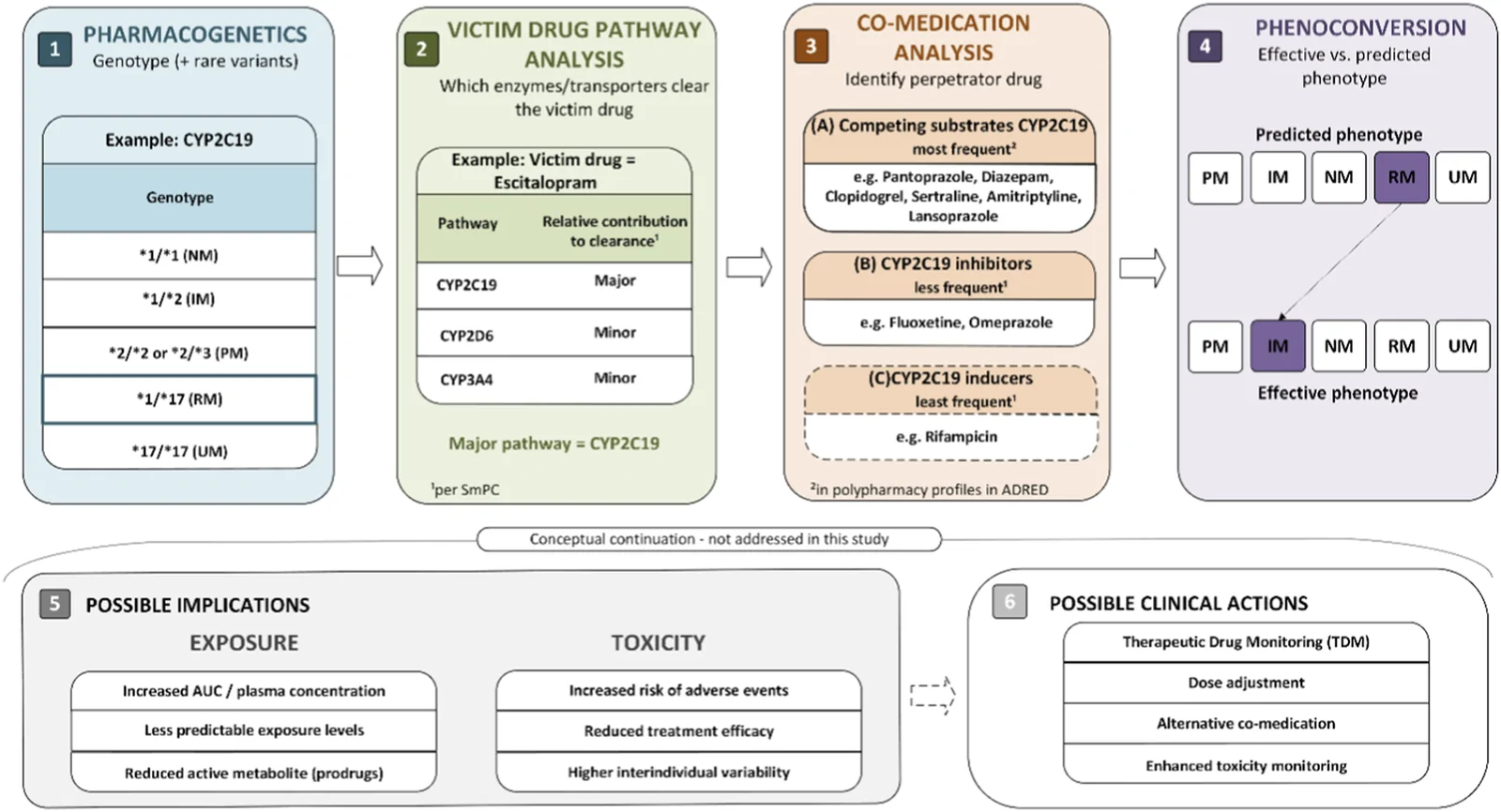
Conceptual framework linking germline pharmacogenetic variation to clinical outcome. The figure outlines the mechanistic chain from genotype through enzyme activity, co-medication and pathway-sharing to drug exposure and clinical outcome, integrating the findings of the present analysis into a systems-pharmacology perspective. 1–4 correspond to the data-supported steps of this analysis, while 5 and 6 outline the conceptual continuation of the pathway and were not addressed empirically in the present cohort. 1 (Pharmacogenetics): Germline variation in genes encoding drug-metabolizing enzymes defines a genotype-predicted phenotype, illustrated here for CYP2C19 and their corresponding metabolizer categories according to CPIC/DPWG guidelines. 2 (Victim drug pathway): The clinical relevance of pharmacogenetic variation depends on the fractional contribution of the polymorphic enzyme to the clearance of the victim drug, shown here for Escitalopram with CYP2C19 as the major clearance pathway. 3 (Co-Medication): Concomitant medication may act on the same enzyme via three routes. It could act as a competing substrate, a pharmacokinetic inhibitor or an inducer. In our polypharmacy cohort (ADRED), substrate mediated pathway sharing emerged as the most frequent scenario across CYP enzymes, whereas inhibitors were less frequent and inducers the least frequent. 4 (potential for phenoconversion): The combined influence of germline variation and comedication may shift the effective enzyme phenotype away from the genotype-predicted phenotype. 5,6 (Possible implications and clinical actions): These panels outline the conceptual downstream consequences of phenoconversion for drug exposure, toxicity and clinical management. Note on the illustrative example. CYP2C19 and escitalopram used throughout 1–4 as a well characterized illustrative example. The framework is not specific to CYP2C19 or to psychiatric medication. It applies to any drug-metabolizing enzyme with actionable pharmacogenetic variation and to any victim drug that depends substantially on the affected pathway, including anticancer drugs as shown in the main text.

This observation is particularly relevant in oncology, where therapies are often administered below the maximum tolerated dose (Denduluri et al., 2015). The maximum tolerated dose is ultimately determined by the occurrence of toxicity, which in turn is influenced by individual drug exposure, metabolic capacity, and drug–drug interactions. Accordingly, pharmacogenetic-guided dosing strategies have been shown to improve treatment individualization and may contribute to optimizing efficacy and safety through more precise estimation of drug exposure (Roncato et al., 2017; Roncato et al., 2018; Roncato et al., 2022).

Our findings indicate that exposure to drugs sharing or modifying pharmacogenetically relevant metabolic pathways is common among patients with polypharmacy. As a consequence, genotype-based predictions alone may not fully reflect the effective metabolic capacity of individual patients.

Several limitations should be considered. First, no genotype information, enzyme activity data, pharmacokinetic parameters, or prospective clinical outcomes were available. Consequently, the present study cannot determine whether phenoconversion actually occurred in individual patients, nor can it quantify its impact on drug exposure, toxicity, or treatment efficacy. Instead, the results should be interpreted as an estimate of the potential metabolic pathway overlap within a clinically relevant polypharmacy setting.

An additional limitation is that individual medication profiles could not be linked to specific anticancer treatment regimens. Future studies combining pharmacogenetic data, detailed anticancer treatment information, and longitudinal medication records will be required to determine the frequency and clinical consequences of pathway overlap and phenoconversion in defined oncology populations.

Regarding the LLM-assisted extraction of metabolic pathways for the individual ADRED drugs, the observed error rate was 6.0%, corresponding to agreement with the manual reference standard in 94.0% of the evaluated cases. Although not directly comparable because of differences in tasks and used models, the observed error rate is within the range suggested by previously reported performance estimates for LLM-based information extraction from pharmaceutical product labels (Gisladottir et al., 2026). The relatively wide 95% confidence interval of 2.1%–16.2% reflects the limited precision of the estimate based on 50 evaluated drugs. Therefore, these findings should be considered preliminary rather than evidence of a universally applicable error rate. Future work should evaluate performance across a broader range of substances, document formats, and pharmacokinetic reporting styles to further establish the robustness and generalizability of the extraction workflow.

## Data Availability

The original contributions presented in the study are included in the article/Supplementary Material, further inquiries can be directed to the corresponding author.
