# Supplementary material for: Estimating metabolic pathway overlap in precision oncology: integrating pharmacogenetics and polypharmacy
Source: Front Pharmacol. 2026 Oct 26;17:1947359. doi: 10.3389/fphar.2026.1947359 (PMC13745669; doi:10.3389/fphar.2026.1947359)
Supplement: Supplementary file 1 [file DataSheet1.pdf]

Supplementary Material

Supplementary Code 1.

The following Python script implements a two-step LLM-assisted pipeline for extracting pharmacokinetic information from German drug product information documents. Both extraction steps used the model vllm/google/gemma-4-31B-it. In Step 1, the model was prompted to extract information on unchanged elimination and CYP-mediated metabolism from raw German SmPC HTML text and to return the results as a structured Markdown summary. In Step 2, a second prompt was used to convert this Markdown summary into a predefined JSON schema validated with Pydantic. The final output includes numerical and textual information on unchanged elimination, exact supporting quotations, and structured CYP involvement entries. Step 1 was run with a temperature of 0.7, whereas Step 2 was run with a temperature of 0.3. The full prompts used in both LLM-based processing steps are provided below. The complete Python script (pk_extraction.py) is available in the associated BWGit repository:

URL: https://bwgit.de/hd-vy353/Phenoconversion_Precision_Oncology

Prompt used in Step 1: HTML to Markdown extraction

The original prompt was provided to the model in German. It is reported below verbatim to ensure reproducibility. An English summary of the prompt purpose is provided before the verbatim prompt.

English summary:

This prompt instructed the model to act as a medical expert and extract pharmacokinetic information from German SmPC documents, focusing on unchanged elimination and CYP-mediated metabolism.

Original prompt:

"""Rolle & Aufgabe:

Du bist ein medizinischer Fachexperte. Deine Aufgabe ist es, pharmakokinetische Daten (Elimination und Metabolismus) zu einer spezifischen Substanz aus einer Fachinformation zu extrahieren.

Vorgehen:

Durchsuche primär Kapitel "5.2 Pharmakokinetische Eigenschaften". Greife nur auf andere Kapitel zurück, wenn dort absolut keine Angaben zu finden sind.

Bei Kombinationspräparaten fokussiere dich auf die hier angegebene Substanz.

Finde Informationen zu zwei Bereichen:

1. Unveränderte Ausscheidung (Elimination): Zu welchem Anteil (in Prozent/Dezimal) wird die Substanz unverändert (renal oder biliär) ausgeschieden?

2. CYP-Metabolismus: Welche Cytochrom-P450-Enzyme (spezifisch: CYP1A2, CYP2B6, CYP2C19, CYP2C8, CYP2C9, CYP2D6, CYP3A) sind am Abbau beteiligt und zu welchen quantitativen Anteilen?

Regeln zur Quantifizierung (Wertebereich 0.0 bis 1.0):

Übersetze textliche Mengenangaben in dezimale Schätzwerte. Nutze folgende Richtwerte als Basis für deine Schätzung:

- "vollständig" / "ausschließlich" = 1.0

- "hauptsächlich" / "überwiegend" / "größtenteils" ≈ 0.8

- "die Hälfte" = 0.5

- "teilweise" / "moderat" ≈ 0.3

- "gering" / "untergeordnet" / "in geringem Ausmaß" ≈ 0.1

- Die Summe der quantitativen Anteile über alle beteiligten CYP-Enzyme darf 1.0 auf keinen Fall überschreiten!

Ausgabeformat (Markdown):

Antworte exakt in dieser Struktur:

Reasoning

[Schreibe hier eine kurze, nachvollziehbare Zusammenfassung deiner Erkenntnisse zu beiden Bereichen. Erkläre anhand der obigen Regeln, wie du auf eventuelle quantitative Werte kommst.]

Unveränderte Elimination

- Anteil numerisch: [Dezimalwert zwischen 0.0 und 1.0. Oder "Nicht verfügbar"]

- Beschreibung: [Deine kurze Zusammenfassung]

- Zitat: "[Das exakte, wörtliche Zitat aus dem Text]"

CYP-Metabolismus

[Für jedes im Text erwähnte CYP-Enzym der obigen Liste einen Block anlegen. Wenn keines erwähnt wird, schreibe "Keine spezifischen CYP-Enzyme genannt."]

- Enzym: [Name]

- Beteiligung: [Beschreibung der Beteiligung und dein geschätzter quantitativer Anteil]

- Zitat: "[Das exakte, wörtliche Zitat]"

Substanz:

{substance}

Fachinformation (Raw HTML):

{html_text}"""

Prompt used in Step 2: Markdown to JSON structuring

English summary:

This prompt instructed the model to act as a data-processing assistant and convert the Markdown extraction generated in Step 1 into a strict JSON format. The prompt specified how to populate the analysis field, how to encode missing information on unchanged elimination. It also instructed the model to mark each enzyme as involved or not involved and to set all descriptive fields to null when an enzyme was not involved.

Original prompt:

"""Du bist ein Datenverarbeitungs-Assistent. Deine einzige Aufgabe ist es, den dir vorliegenden Markdown-Text in ein striktes JSON-Format zu übersetzen.

Regeln:

1. Nutze die "Analyse" aus dem Text für das "analysis" Feld.

2. Wenn bei der unveränderten Elimination kein Wert gefunden wurde, setze "unchanged_elimination_numeric" auf -1.0 und die Textfelder auf "unknown".

3. Du musst zwingend alle 7 Enzyme in die "cyp_entries" Liste aufnehmen: ["CYP_1A2", "CYP_2B6", "CYP_2C19", "CYP_2C8", "CYP_2C9", "CYP_2D6", "CYP_3A"].

4. Setze "involved" auf 1, wenn das Enzym im Text als beteiligt genannt wird, ansonsten auf 0.

5. Wenn "involved" = 0 ist, setze "involved_quantified", "description" und "exact_cite" zwingend auf null.

Hier ist der zu strukturierende Markdown-Text:

{markdown_text}"""

Supplementary Figures:

Supplementary Figure S1. Metabolic pathways of n = 839 individual ADRED drugs (Table S1)

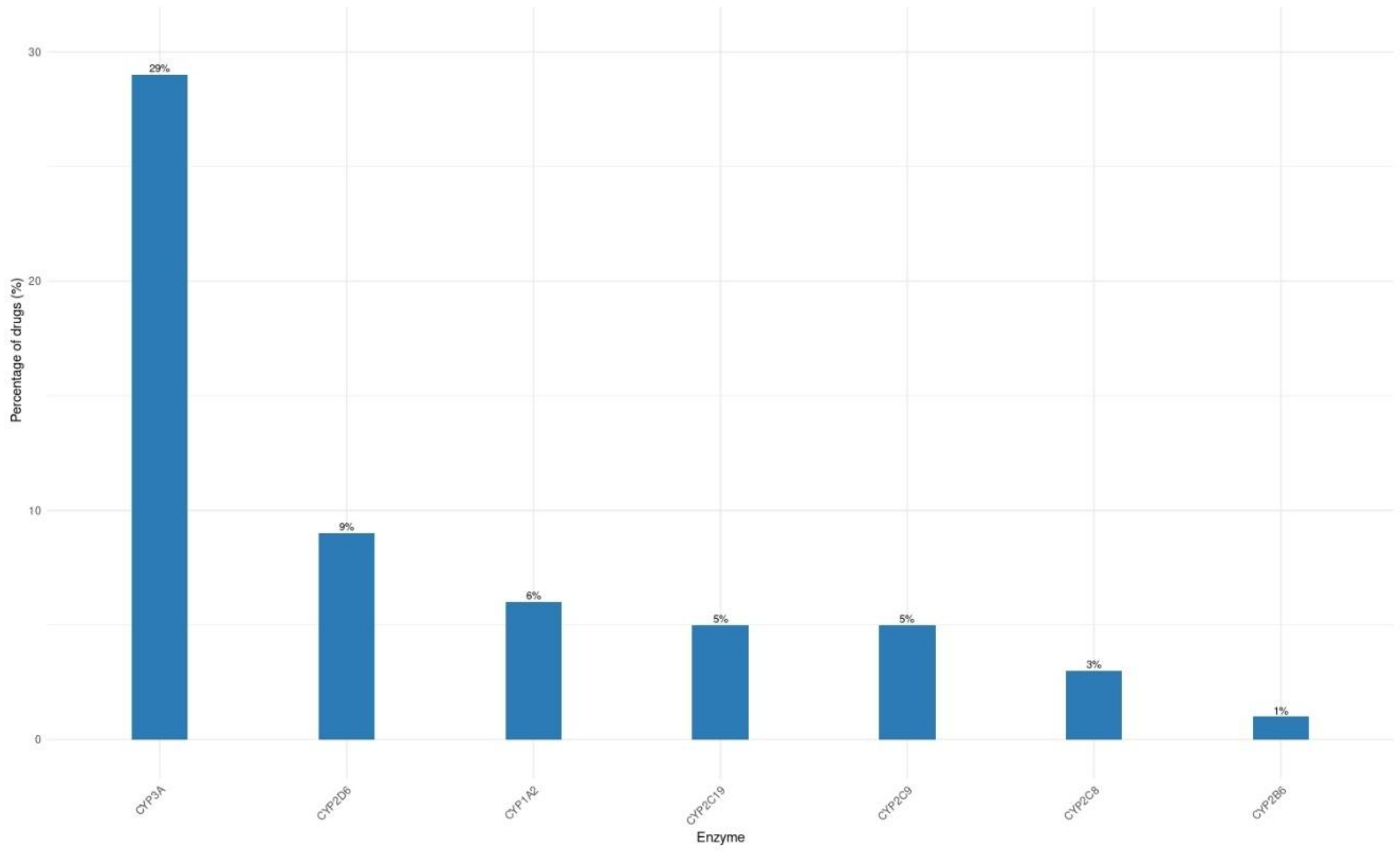

Supplementary Tables:

Table S1: ADRED cases containing at least one FDA-labeled CYP inhibitor or inducer by CYP enzyme and interaction strength.

**CYP enzyme**<br><br>**total/weak/moderate/strong**	**General polypharmacy cohort** (ADRED):<br><br>n = 7400	**Gastrointestinal oncology cohort:**<br><br>n = 567
**CYP1A2**_inducer	0.2% (12) / 0.0% (0) / 0.2% (12) / 0.0% (0)	0.0% (0) / 0.0% (0) / 0.0% (0) / 0.0% (0)
**CYP1A2**_inhibitor	12.9% (952) / 12.1% (895) / 0.8% (62) / 0.0% (0)	15.5% (88) / 11.1% (63) / 5.1% (29) / 0.0% (0)
**CYP2B6**_inducer	0.6% (47) / 0.0% (0) / 0.0% (0) / 0.6% (47)	0.7% (4) / 0.0% (0) / 0.2% (1) / 0.5% (3)
**CYP2B6**_inhibitor	6.8% (502) / 6.8% (502) / 0.0% (0) / 0.0% (0)	12.5% (71) / 12.5% (71) / 0.0% (0) / 0.0% (0)
**CYP2C8**_inducer	0.0% (0) / 0.0% (0) / 0.0% (0) / 0.0% (0)	0.0% (0) / 0.0% (0) / 0.0% (0) / 0.0% (0)
**CYP2C8**_inhibitor	9.0% (668) / 2.1% (159) / 6.9% (513) / 0.0% (1)	17.6% (100) / 5.8% (33) / 12.7% (72) / 0.0% (0)
**CYP2C9**_inducer	0.9% (67) / 0.8% (58) / 0.1% (9) / 0.0% (0)	0.9% (5) / 0.7% (4) / 0.2% (1) / 0.0% (0)
**CYP2C9**_inhibitor	2.6% (196) / 0.7% (49) / 2.0% (149) / 0.0% (0)	4.1% (23) / 1.6% (9) / 2.5% (14) / 0.0% (0)
**CYP2C19**_inducer	0.2% (16) / 0.0% (0) / 0.2% (16) / 0.0% (0)	0.4% (2) / 0.0% (0) / 0.4% (2) / 0.0% (0)
**CYP2C19**_inhibitor	4.0% (298) / 3.5% (260) / 0.1% (4) / 0.5% (34)	4.2% (24) / 3.5% (20) / 0.2% (1) / 0.5% (3)
**CYP2D6**_inducer	0.0% (0) / 0.0% (0) / 0.0% (0) / 0.0% (0)	0.0% (0) / 0.0% (0) / 0.0% (0) / 0.0% (0)
**CYP2D6**_inhibitor	8.1% (600) / 5.1% (378) / 2.6% (190) / 0.6% (43)	7.6% (43) / 5.6% (32) / 1.2% (7) / 0.7% (4)
**CYP3A**_inducer	1.0% (75) / 0.0% (1) / 0.1% (11) / 0.9% (64)	1.1% (6) / 0.2% (1) / 0.2% (1) / 0.7% (4)
**CYP3A**_inhibitor	6.8% (500) / 3.9% (286) / 2.6% (193) / 0.4% (30)	15.9% (90) / 7.1% (40) / 6.9% (39) / 2.6% (15)

Table S2: Table of n = 839 individual ADRED drugs, used for analysis.

**No.**	**Active substance**
1	Abacavir
2	Abatacept
3	Abirateron
4	Acalabrutinib

5	Acarbose
6	Acemetacin
7	Acetazolamid
8	Acetylcystein
9	Acetyldigoxin
10	Acetylsalicylsäure
11	Aciclovir
12	Acitretin
13	Aclidiniumbromid
14	Adapalen
15	Agomelatin
16	Albendazol
17	Alectinib
18	Alendronsäure
19	Alfacalcidol
20	Alfacalcidol (vitamin d)
21	Alfuzosin
22	Algeldrat
23	Allopurinol
24	Alpha-liponsäure (thioctsäure)
25	Alprazolam
26	Amantadin
27	Ambrisentan
28	Ambroxol
29	Amidotrizoesäure
30	Amikacin
31	Amilorid
32	Amiodaron
33	Amisulprid
34	Amitriptylin
35	Amlodipin
36	Amoxicillin
37	Amphotericin b
38	Ampicillin
39	Anagrelid
40	Anastrozol
41	Apixaban
42	Apraclonidin
43	Apremilast
44	Aprepitant
45	Arginin
46	Aripiprazol
47	Articain
48	Ascorbinsäure
49	Ascorbinsäure (vitamin c)
50	Atenolol

51	Atorvastatin
52	Atropin
53	Axitinib
54	Azacitidin
55	Azathioprin
56	Azithromycin
57	Baclofen
58	Baricitinib
59	Bcg-impfstoff
60	Bcg-lebend-impfstoff; stamm rivm
61	Beclometason
62	Bemetizid
63	Bempedoinsäure
64	Benazepril
65	Bendamustin
66	Benserazid
67	Benzbromaron
68	Benzocain
69	Benzoylperoxid
70	Benzydamin
71	Benzylpenicillin-benzathin
72	Beta-acetyldigoxin
73	Betahistin
74	Betamethason
75	Betaxolol
76	Bezafibrat
77	Bicalutamid
78	Bictegravir
79	Bimatoprost
80	Binimetinib
81	Biperiden
82	Bisacodyl
83	Bisoprolol
84	Bleomycin
85	Bortezomib
86	Bosentan
87	Bosutinib
88	Brimonidin
89	Brinzolamid
90	Brivaracetam
91	Brivudin
92	Bromazepam
93	Bromelain
94	Bromelaine
95	Bromocriptin
96	Brotizolam

97	Budesonid
98	Buprenorphin
99	Bupropion
100	Buserelin
101	Buspiron
102	Butylscopolamin
103	Cabazitaxel
104	Cabergolin
105	Cabozantinib
106	Cafedrin
107	Calcipotriol
108	Calcitriol
109	Calcitriol (vitamin d)
110	Calcium
111	Candesartan
112	Cannabidiol
113	Cannabis
114	Cannabis sativa l.
115	Capecitabin
116	Captopril
117	Carbamazepin
118	Carbidopa
119	Carbimazol
120	Carboplatin
121	Carfilzomib
122	Carmustin
123	Carnitin
124	Carvedilol
125	Cefaclor
126	Cefadroxil
127	Cefazolin
128	Cefixim
129	Cefotaxim
130	Cefpodoxim
131	Ceftazidim
132	Ceftriaxon
133	Cefuroxim
134	Celecoxib
135	Ceritinib
136	Certoparin
137	Cetirizin
138	Chinin
139	Chloralhydrat
140	Chlorambucil
141	Chlordiazepoxid
142	Chlorhexidin

143	Chlormadinon
144	Chlorphenamin
145	Chlorprothixen
146	Chlortalidon
147	Choriongonadotrophin
148	Ciclesonid
149	Ciclopirox
150	Ciclosporin
151	Cilostazol
152	Cimetidin
153	Cinacalcet
154	Cinchocain
155	Cinnarizin
156	Ciprofloxacin
157	Cisplatin
158	Citalopram
159	Citronensäure
160	Citrus aurantium l. ssp. aurantium
161	Cladribin
162	Clarithromycin
163	Clavulansäure
164	Clemastin
165	Clindamycin
166	Clioquinol
167	Clobazam
168	Clobetasol
169	Clomethiazol
170	Clomifen
171	Clomipramin
172	Clonazepam
173	Clonidin
174	Clopamid
175	Clopidogrel
176	Clorazepat
177	Clotrimazol
178	Clozapin
179	Co-trimoxazol
180	Cobimetinib
181	Codein
182	Coffein
183	Colchicin
184	Colchicum
185	Colecalciferol
186	Colecalciferol (vitamin d)
187	Colestyramin
188	Cortison

189	Crataegus
190	Crizotinib
191	Cromoglicinsäure
192	Curcuma
193	Cyanocobalamin
194	Cyanocobalamin (vitamin b12
195	Cyanocobalamin (vitamin b12)
196	Cyanocobalaminum
197	Cyclophosphamid
198	Cyproteron
199	Cytarabin
200	Cytomegalievirus-immunglobulin
201	D-glucosamin vom schalentier
202	Dabigatran
203	Dabigatran etexilat
204	Dacarbazin
205	Dalteparin
206	Dantrolen
207	Dapagliflozin
208	Dapoxetin
209	Dapson
210	Darbepoetin alfa
211	Darifenacin
212	Darunavir
213	Dasatinib
214	Daunorubicin
215	Decitabin
216	Deferasirox
217	Degarelix
218	Desfesoterodin
219	Desfluran
220	Desloratadin
221	Desmopressin
222	Desogestrel
223	Dexamethason
224	Dexibuprofen
225	Dexketoprofen
226	Dextromethorphan
227	Diazepam
228	Diclofenac
229	Dienogest
230	Diflucortolon
231	Digitoxin
232	Digoxin
233	Dihydralazin
234	Dihydrocodein

235	Dikaliumclorazepat
236	Diltiazem
237	Dimenhydrinat
238	Dimethylfumarat
239	Dimeticon
240	Dimetinden
241	Diphenhydramin
242	Diphtherie-adsorbat-impfstoff
243	Dl-α-tocopherol
244	Docetaxel
245	Dolutegravir
246	Dolutegravir-natrium
247	Domperidon
248	Donepezil
249	Dorzolamid
250	Doxazosin
251	Doxepin
252	Doxorubicin
253	Doxycyclin
254	Doxylamin
255	Dronedaron
256	Drospirenon
257	Dulaglutid
258	Duloxetin
259	Dutasterid
260	Dydrogesteron
261	Ebastin
262	Econazol
263	Edoxaban
264	Efavirenz
265	Eisen
266	Eisen ii
267	Eltrombopag
268	Empagliflozin
269	Emtricitabin
270	Enalapril
271	Encorafenib
272	Enoxaparin
273	Entacapon
274	Entecavir
275	Enzalutamid
276	Ephedrin
277	Epinephrin
278	Epinephrin (adrenalin)
279	Epirubicin
280	Eplerenon

281	Epoetin alfa
282	Epoetin zeta
283	Eprosartan
284	Eribulin
285	Ertapenem
286	Ertugliflozin
287	Erythromycin
288	Escitalopram
289	Esketamin
290	Eslicarbazepin
291	Esomeprazol
292	Estradiol
293	Estriol
294	Eszopiclon
295	Etanercept
296	Ethambutol
297	Ethinylestradiol
298	Etilefrin
299	Etonogestrel
300	Etoposid
301	Etoricoxib
302	Eucalyptus
303	Eucalyptusöl
304	Everolimus
305	Exemestan
306	Exenatid
307	Ezetimib
308	Famotidin
309	Fampridin
310	Febuxostat
311	Felodipin
312	Fenofibrat
313	Fenoterol
314	Fentanyl
315	Fesoterodin
316	Fexofenadin
317	Filgrastim
318	Finasterid
319	Fingolimod
320	Flecainid
321	Flucloxacillin
322	Fluconazol
323	Fludarabin
324	Fludrocortison
325	Flumetason
326	Flunarizin

327	Flunisolid
328	Fluocinolon acetonid
329	Fluorouracil
330	Fluoxetin
331	Flupentixol
332	Fluphenazin
333	Fluprednidien
334	Flurbiprofen
335	Fluspirilen
336	Fluticason
337	Fluticasonfuroat
338	Fluvastatin
339	Fluvoxamin
340	Folinsäure
341	Folsäure
342	Fondaparinux
343	Formoterol
344	Fosaprepitant
345	Fosfomycin
346	Fosinopril
347	Fulvestrant
348	Furosemid
349	Fusidinsäure
350	Gabapentin
351	Gadotersäure
352	Galantamin
353	Gelbfieber-lebend-impfstoff (pcec); stamm 17d-204
354	Gemcitabin
355	Gemfibrozil
356	Gentamicin
357	Ginkgo
358	Glatiramer
359	Glatirameracetat
360	Glibenclamid
361	Glimepirid
362	Gliquidon
363	Glycerol
364	Glyceroltrinitrat
365	Glyceroltrinitrat (nitroglycerin)
366	Glycopyrronium
367	Glycopyrronium bromid
368	Glycopyrroniumbromid
369	Goserelin
370	Granisetron
371	Guaifenesin

372	Haloperidol
373	Harnstoff
374	Hepar suis
375	Heparin
376	Histaminum
377	Hydralazin
378	Hydrochlorothiazid
379	Hydrocortison
380	Hydromorphon
381	Hydroxocobalamin
382	Hydroxocobalamin (vitamin b12)
383	Hydroxycarbamid
384	Hydroxychloroquin
385	Hydroxyzin
386	Ibandronsäure
387	Ibrutinib
388	Ibuprofen
389	Icatibant
390	Icosapent
391	Idarubicin
392	Idelalisib
393	Ifosfamid
394	Imatinib
395	Imipramin
396	Indacaterol
397	Indapamid
398	Indometacin
399	Insulin (human)
400	Insulin aspart
401	Insulin aspart-isophan
402	Insulin degludec
403	Insulin detemir
404	Insulin glargin
405	Insulin glulisin
406	Insulin lispro
407	Insulin lispro-isophan
408	Insulin-isophan
409	Insulin-isophan (human)
410	Interferon beta-1a
411	Interferon beta-1b
412	Iod
413	Iopamidol
414	Iopromid
415	Ipratropiumbromid
416	Irbesartan
417	Irinotecan

418	Isoconazol
419	Isoniazid
420	Isosorbidmononitrat
421	Itraconazol
422	Ivabradin
423	Ivermectin
424	Ixazomib
425	Kali
426	Kalium
427	Kaliumiodid
428	Ketoprofen
429	Ketotifen
430	L-methionin
431	Lacosamid
432	Lactulose
433	Lamivudin
434	Lamotrigin
435	Lansoprazol
436	Lanthan
437	Lapatinib
438	Larotrectinib sulfat
439	Latanoprost
440	Leflunomid
441	Lenalidomid
442	Lenograstim
443	Lenvatinib
444	Lercanidipin
445	Letrozol
446	Leuprorelin
447	Levetiracetam
448	Levocarnitin
449	Levocetirizin
450	Levodopa
451	Levofloxacin
452	Levomepromazin
453	Levomethadon
454	Levonorgestrel
455	Levothyroxin
456	Levothyroxin-natrium
457	Lidocain
458	Linezolid
459	Liothyronin
460	Liothyronin-natrium
461	Lipegfilgrastim
462	Liraglutid
463	Lisinopril

464	Lithium
465	Lomustin
466	Loperamid
467	Loratadin
468	Lorazepam
469	Lormetazepam
470	Losartan
471	Loteprednol
472	Lovastatin
473	Macrogol
474	Magaldrat
475	Magnesium
476	Maprotilin
477	Mebeverin
478	Medizinische kohle
479	Medroxyprogesteron
480	Megestrol
481	Melatonin
482	Meloxicam
483	Melperon
484	Melphalan
485	Memantin
486	Mepivacain
487	Meropenem
488	Mesalazin
489	Mesna
490	Metamizol
491	Metformin
492	Methadon
493	Methantheliniumbromid
494	Methionin
495	Methocarbamol
496	Methotrexat
497	Methyldopa
498	Methyldopa (linksdrehend)
499	Methylergometrin
500	Methylphenidat
501	Methylprednisolon
502	Methylprednisolonaceponat
503	Metildigoxin
504	Metoclopramid
505	Metoprolol
506	Metronidazol
507	Mexiletin
508	Mianserin
509	Miconazol

510	Midazolam
511	Midodrin
512	Midostaurin
513	Milnacipran
514	Minocyclin
515	Minoxidil
516	Mirtazapin
517	Misoprostol
518	Mitomycin
519	Mitotan
520	Mitoxantron
521	Mizolastin
522	Moclobemid
523	Molsidomin
524	Mometason
525	Monoblast
526	Montelukast
527	Morphin
528	Moxifloxacin
529	Moxonidin
530	Mycophenolsäure
531	Nadroparin
532	Nalmefen
533	Naloxegol
534	Naloxon
535	Naltrexon
536	Naproxen
537	Naratriptan
538	Natriumbicarbonat
539	Natriumchlorid
540	Natriumchlorit
541	Natriumfluorid
542	Natriumhydrogencarbonat
543	Natriumperchlorat
544	Natriumpicosulfat
545	Nebivolol
546	Neomycin
547	Nepafenac
548	Nicergolin
549	Nicotin
550	Nicotina
551	Nicotinamid
552	Nicotinsäure
553	Nifedipin
554	Nintedanib
555	Niraparib

556	Nitrazepam
557	Nitrendipin
558	Nitrofurantoin
559	Nitroxolin
560	Norelgestromin
561	Norepinephrin
562	Norethisteron
563	Noscapin
564	Nystatin
565	Ofloxacin
566	Olanzapin
567	Olmesartan
568	Olodaterol
569	Omega-3-fettsäure
570	Omega-3-fettsäuren
571	Omeprazol
572	Ondansetron
573	Opicapon
574	Opipramol
575	Ornithin
576	Oseltamivir
577	Oxaceprol
578	Oxaliplatin
579	Oxazepam
580	Oxcarbazepin
581	Oxybuprocain
582	Oxybutynin
583	Oxycodon
584	Oxytetracyclin
585	Paclitaxel
586	Palbociclib
587	Paliperidon
588	Pamidronsäure
589	Panax ginseng
590	Panax ginseng c. a. meyer
591	Pankreas-pulver vom schwein
592	Pankreatin (schwein)
593	Pantoprazol
594	Paracetamol
595	Paricalcitol
596	Paroxetin
597	Passiflora incarnata
598	Passiflora incarnata d4
599	Patiromercalcium
600	Pazopanib
601	Peg-epoetin beta

602	Pegaspargase
603	Pegfilgrastim
604	Peginterferon beta-1a
605	Pemetrexed
606	Penicillin
607	Pentaerythrityltetranitrat
608	Pentoxifyllin
609	Pentoxyverin
610	Perampanel
611	Perazin
612	Perindopril
613	Pethidin
614	Phenazon
615	Phenobarbital
616	Phenoxybenzamin
617	Phenoxymethylpenicillin
618	Phenprocoumon
619	Phenylalani
620	Phenylalanin
621	Phenylephrin
622	Phenytoin
623	Phospholipide
624	Phytomenadion
625	Phytomenadion (vitamin k)
626	Pilocarpin
627	Pioglitazon
628	Pipamperon
629	Piperacillin
630	Piracetam
631	Pirenzepin
632	Piretanid
633	Pirfenidon
634	Piribedil
635	Piritramid
636	Piroxicam
637	Pivmecillinam
638	Polymyxin-b
639	Polystyrolsulfonat
640	Pomalidomid
641	Posaconazol
642	Povidon
643	Povidon-iod
644	Pramipexol
645	Prasteron
646	Prasugrel
647	Pravastatin

648	Prazepam
649	Prednicarbat
650	Prednisolon
651	Prednison
652	Pregabalin
653	Pridinol
654	Primidon
655	Procarbazin
656	Progesteron
657	Proglumetacin
658	Promethazin
659	Propafenon
660	Propiverin
661	Propofol
662	Propranolol
663	Propylthiouracil
664	Prothipendyl
665	Prucaloprid
666	Pseudoephedrin
667	Pyrazinamid
668	Pyridostigmin
669	Pyridoxin
670	Pyridoxin (vitamin b6)
671	Pyrimethamin
672	Quetiapin
673	Quinagolid
674	Quinapril
675	Rabeprazol
676	Racecadotril
677	Raloxifen
678	Ramipril
679	Ranolazin
680	Rasagilin
681	Repaglinid
682	Reproterol
683	Retinol
684	Retinol (vitamin a)
685	Ribavirin
686	Ribociclib
687	Rifampicin
688	Rifaximin
689	Riluzol
690	Riociguat
691	Risedronsäure
692	Risperidon
693	Ritonavir

694	Rivaroxaban
695	Rivastigmin
696	Rizatriptan
697	Roflumilast
698	Romiplostim
699	Ropinirol
700	Ropivacain
701	Rosuvastatin
702	Rotigotin
703	Roxithromycin
704	Rupatadin
705	Ruxolitinib
706	Saccharomyces boulardii
707	Sacubitril
708	Safinamid
709	Salbutamol
710	Salicylsäure
711	Salmeterol
712	Sauerstoff
713	Saxagliptin
714	Scopolamin
715	Selen
716	Semaglutid
717	Sertralin
718	Sevelamer
719	Sildenafil
720	Silodosin
721	Silymarin
722	Simeticon
723	Simvastatin
724	Siponimod
725	Sirolimus
726	Sitagliptin
727	Solifenacin
728	Sorafenib
729	Sorbitol
730	Sotalol
731	Spironolacton
732	Sucralfat
733	Sulbactam
734	Sulfadiazin
735	Sulfamethoxazol
736	Sulfasalazin
737	Sulpirid
738	Sultamicillin
739	Sumatriptan

740	Sunitinib
741	Tacrolimus
742	Tadalafil
743	Tafluprost
744	Talimogen laherparepvec
745	Tamoxifen
746	Tamsulosin
747	Tapentadol
748	Tazobactam
749	Telmisartan
750	Temazepam
751	Temozolomid
752	Temsirolimus
753	Tenofovir disoproxil
754	Tenofoviralafenamid
755	Tenofovirdisoproxil
756	Terazosin
757	Terbinafin
758	Teriflunomid
759	Teriparatid
760	Testosteron
761	Tetanus-adsorbat-impfstoff
762	Tetrabenazin
763	Tetracyclin
764	Tetryzolin
765	Theodrenalin
766	Theophyllin
767	Thiamazol
768	Thiaminium-kation
769	Thioridazin
770	Thiotepa
771	Tianeptin
772	Tiaprid
773	Tibolon
774	Ticagrelor
775	Tilidin
776	Timolol
777	Tinzaparin
778	Tiotropiumbromid
779	Tipiracil
780	Tizanidin
781	Tocopherol (vitamin e)
782	Tofacitinib
783	Tolcapon
784	Tolperison
785	Tolterodin

786	Tolvaptan
787	Topiramat
788	Topotecan
789	Torasemid
790	Tramadol
791	Trametinib
792	Tranexamsäure
793	Tranylcypromin
794	Travoprost
795	Trazodon
796	Tretinoin
797	Triamcinolon
798	Triamcinolon acetonid
799	Triamteren
800	Triclosan
801	Trifluridin
802	Trimethoprim
803	Trimipramin
804	Tropicamid
805	Trospium
806	Tryptophan
807	Umeclidiniumbromid
808	Urapidil
809	Ursodeoxycholsäure
810	Valaciclovir
811	Valeriana officinalis d4
812	Valganciclovir
813	Valproinsäure
814	Valsartan
815	Vancomycin
816	Vardenafil
817	Vemurafenib
818	Venetoclax
819	Venlafaxin
820	Verapamil
821	Vilanterol
822	Vildagliptin
823	Vinblastin
824	Vincristin
825	Vindesin
826	Vinflunin
827	Vinorelbin
828	Voriconazol
829	Warfarin
830	Xipamid
831	Xylometazolin

832	Zink
833	Ziprasidon
834	Zitronensäure
835	Zoledronsäure
836	Zolpidem
837	Zonisamid
838	Zopiclon
839	Zuclopenthixol

Table S3: Comparison of observed CYP pathway involvement between the general polypharmacy cohort and GIT oncology cohort.

Enzyme	General polypharmacy cohort n/N (%)	GIT oncology cohort n/N (%)	Prevalence difference pp (95% CI)	Involvement ratio (95% CI)	Fisher p	Holm p
CYP1A2	2484/7400 (33.6%)	265/567 (46.7%)	+13.2 (+9.0 to +17.4)	1.39 (1.27-1.53)	4.997E-10	2.499E-09
CYP3A	6201/7400 (83.8%)	528/567 (93.1%)	+9.3 (+6.8 to +11.3)	1.11 (1.08-1.14)	1.784E-10	1.070E-09
CYP2B6	787/7400 (10.6%)	95/567 (16.8%)	+6.1 (+3.2 to +9.5)	1.58 (1.30-1.91)	2.107E-05	5.871E-05
CYP2C9	2943/7400 (39.8%)	247/567 (43.6%)	+3.8 (-0.4 to +8.1)	1.10 (0.99-1.21)	0.083	0.083
CYP2C8	1706/7400 (23.1%)	177/567 (31.2%)	+8.2 (+4.4 to +12.2)	1.35 (1.19-1.54)	1.957E-05	5.871E-05
CYP2C19	4011/7400 (54.2%)	407/567 (71.8%)	+17.6 (+13.6 to +21.3)	1.32 (1.25-1.40)	1.395E-16	9.762E-16
CYP2D6	4247/7400 (57.4%)	393/567 (69.3%)	+11.9 (+7.8 to +15.7)	1.21 (1.14-1.28)	2.287E-08	9.146E-08

Table S4: Comparison of CYP enzyme involvement in oncologic and ADRED drugs

Drug involvement is reported as a percentage. Odds ratios compare oncologic drugs with ADRED drugs. Holm-adjusted p-values correct for multiple testing.

Enzyme	Oncologic drug involvement [%]	ADRED drug involvement [%]	odds_ratio	p_value	holm_p_value
CYP1A2	2.9	5.9	0.5	0.716	1.0
CYP2B6	0.0	1.3	0.0	1.0	1.0
CYP2C19	5.7	5.5	1.1	1.0	1.0
CYP2C8	11.4	2.7	4.6	0.019	0.116
CYP2C9	11.4	5.1	2.4	0.111	0.557
CYP2D6	8.6	8.8	1.0	1.0	1.0
CYP3A	68.6	29.4	5.2	< 0.001	< 0.001

Table S5: CYP inducer and inhibitor prevalence relative to substrate prevalence in the general polypharmacy and oncology polypharmacy cohorts. Gen_poly, general polypharmacy cohort; Onc_poly, oncology polypharmacy cohort; substrate_n, number of cases with at least one medication involving the respective CYP enzyme; role_n, number of cases with at least one weak, moderate, or strong FDA-classified inducer or inhibitor of the respective CYP enzyme, as specified in the comparison column; both_n, number of cases with both at least one substrate and at least one inducer or inhibitor of the same CYP enzyme; pp, percentage points; prevalence_difference_pp, role prevalence minus substrate prevalence, expressed in percentage points; prevalence_ratio_percent, role prevalence expressed as a percentage of the corresponding substrate prevalence; NE, not estimable.

cohort	enzyme	comparison	substrate_n	role_n	both_n	prevalence_difference_pp (95% CI)	prevalence_ratio_percent (95% CI)	mcnemar_p	holm_p
Gen_poly	CYP1A2	inducer_vs_substrate	2484	12	3	-33.4 (-34.5 to -32.3)	0.5 (0.2 to 0.8)	< 0.001	< 0.001
Gen_poly	CYP1A2	inhibitor_vs_substrate	2484	952	370	-20.7 (-22.0 to -19.4)	38.3 (35.8 to 41.0)	< 0.001	< 0.001
Gen_poly	CYP2B6	inducer_vs_substrate	787	47	4	-10.0 (-10.7 to -9.3)	6.0 (4.3 to 7.8)	< 0.001	< 0.001
Gen_poly	CYP2B6	inhibitor_vs_substrate	787	502	499	-3.9 (-4.3 to -3.4)	63.8 (60.4 to 67.1)	< 0.001	< 0.001
Gen_poly	CYP2C8	inducer_vs_substrate	1706	0	0	-23.1 (-24.0 to -22.1)	0.0 (NE)	< 0.001	< 0.001
Gen_poly	CYP2C8	inhibitor_vs_substrate	1706	668	174	-14.0 (-15.2 to -12.9)	39.2 (36.0 to 42.5)	< 0.001	< 0.001
Gen_poly	CYP2C9	inducer_vs_substrate	2943	67	27	-38.9 (-40.0 to -37.7)	2.3 (1.7 to 2.8)	< 0.001	< 0.001
Gen_poly	CYP2C9	inhibitor_vs_substrate	2943	196	115	-37.1 (-38.3 to -36.0)	6.7 (5.8 to 7.6)	< 0.001	< 0.001
Gen_poly	CYP2C19	inducer_vs_substrate	4011	16	9	-54.0 (-55.1 to -52.8)	0.4 (0.2 to 0.6)	< 0.001	< 0.001
Gen_poly	CYP2C19	inhibitor_vs_substrate	4011	298	279	-50.2 (-51.3 to -49.0)	7.4 (6.6 to 8.3)	< 0.001	< 0.001
Gen_poly	CYP2D6	inducer_vs_substrate	4247	0	0	-57.4 (-58.5 to -56.3)	0.0 (NE)	< 0.001	< 0.001
Gen_poly	CYP2D6	inhibitor_vs_substrate	4247	600	476	-49.3 (-50.5 to -48.0)	14.1 (13.1 to 15.2)	< 0.001	< 0.001
Gen_poly	CYP3A	inducer_vs_substrate	6201	75	73	-82.8 (-83.6 to -81.9)	1.2 (0.9 to 1.5)	< 0.001	< 0.001
Gen_poly	CYP3A	inhibitor_vs_substrate	6201	500	483	-77.0 (-78.0 to -76.1)	8.1 (7.4 to 8.7)	< 0.001	< 0.001
Onc_poly	CYP1A2	inducer_vs_substrate	265	0	0	-46.7 (-50.8 to -42.7)	0.0 (NE)	< 0.001	< 0.001
Onc_poly	CYP1A2	inhibitor_vs_substrate	265	88	45	-31.2 (-36.2 to -26.3)	33.2 (26.6 to 40.4)	< 0.001	< 0.001
Onc_poly	CYP2B6	inducer_vs_substrate	95	4	3	-16.0 (-19.2 to -13.1)	4.2 (1.0 to 8.9)	< 0.001	< 0.001
Onc_poly	CYP2B6	inhibitor_vs_substrate	95	71	70	-4.2 (-6.0 to -2.6)	74.7 (65.4 to 83.5)	< 0.001	< 0.001
Onc_poly	CYP2C8	inducer_vs_substrate	177	0	0	-31.2 (-35.1 to -27.5)	0.0 (NE)	< 0.001	< 0.001
Onc_poly	CYP2C8	inhibitor_vs_substrate	177	100	27	-13.6 (-18.7 to -8.5)	56.5 (44.8 to 70.2)	< 0.001	< 0.001
Onc_poly	CYP2C9	inducer_vs_substrate	247	5	3	-42.7 (-46.9 to -38.4)	2.0 (0.4 to 4.0)	< 0.001	< 0.001
Onc_poly	CYP2C9	inhibitor_vs_substrate	247	23	8	-39.5 (-43.9 to -35.1)	9.3 (5.8 to 13.4)	< 0.001	< 0.001
Onc_poly	CYP2C19	inducer_vs_substrate	407	2	1	-71.4 (-75.1 to -67.7)	0.5 (0.0 to 1.3)	< 0.001	< 0.001
Onc_poly	CYP2C19	inhibitor_vs_substrate	407	24	24	-67.5 (-71.3 to -63.7)	5.9 (3.7 to 8.3)	< 0.001	< 0.001
Onc_poly	CYP2D6	inducer_vs_substrate	393	0	0	-69.3 (-73.0 to -65.4)	0.0 (NE)	< 0.001	< 0.001
Onc_poly	CYP2D6	inhibitor_vs_substrate	393	43	37	-61.7 (-66.0 to -57.5)	10.9 (7.9 to 14.2)	< 0.001	< 0.001
Onc_poly	CYP3A	inducer_vs_substrate	528	6	6	-92.1 (-94.2 to -89.8)	1.1 (0.4 to 2.1)	< 0.001	< 0.001
Onc_poly	CYP3A	inhibitor_vs_substrate	528	90	89	-77.2 (-80.6 to -73.7)	17.0 (13.8 to 20.3)	< 0.001	< 0.001
